# Tunable subwavelength ultrasound focusing in mesoscale spherical lenses using liquid mixtures

**DOI:** 10.1038/s41598-019-50019-0

**Published:** 2019-09-16

**Authors:** Sergio Pérez-López, José Miguel Fuster, Igor V. Minin, Oleg V. Minin, Pilar Candelas

**Affiliations:** 1 Centro de Tecnologías Físicas, Universitat Politècnica de València, València, 46022 Spain; 2 Departamento de Comunicaciones, Universitat Politècnica de València, València, 46022 Spain; 3 Tomsk Polytechnic University, 36 Lenin Avenue, Tomsk, 634050 Russia; 4 Tomsk State University, 30 Lenin Avenue, Tomsk, 634050 Russia

**Keywords:** Acoustics, Applied physics

## Abstract

In this work, we present a configurable spherical lens for underwater focusing applications, which consists on a hollow ABS container filled with a liquid mixture. Two miscible liquids with different sound speeds are required to implement this novel configurable lens. We show that by adjusting the mixing ratio between the volumes of both liquids, the sound speed of the liquid mixture can be accurately selected. This results in a modification of the acoustic jet properties and a continuous tuning on the lens focal length. This procedure can be fully automatized providing a dynamic control mechanism that can shift the lens focal length to any desired value inside a continuous range in both directions. Depending on the acoustic properties of the selected liquids, subwavelength resolution or even beyond the diffraction limit resolution can be achieved. We provide experimental measurements for ethanol-water mixtures achieving subwavelength resolution for a certain focal length ranging between 34.6 and 42.8 mm.

## Introduction

Photonic nanojets have been a hot topic among the scientific community in the past years due to their high focusing capabilities^1–3^. Nanojets can achieve subwavelength or even beyond the diffraction limit resolution, also known as superfocusing. In acoustics, analogous studies have been presented^4–6^ and acoustic jets have been experimentally demonstrated using a solid Rexolite sphere immersed in water^7^ and a liquid cylinder shell filled with perfluorinated oil and surrounded by ethanol^8^. Some approaches to achieve acoustic subwavelength focusing include acoustic metasurfaces with coiling-up space structures, Helmholtz resonators or membrane-type structures^9–12^. Other alternatives based on Super Oscillatory Acoustic Lenses (SOAL)^13^ or ultra-compact planar metasurfaces have been implemented with subwavelength slits^14,15^. However, the manufacturing stage of these kind of devices demands a complete 3D design and optimization of the structure when 3D focusing is required. In this sense, spherical acoustic lenses are simpler alternatives that can provide 3D superfocusing in the near field for a fixed focal distance.

Having accurate control of the focal length and achieving subwavelength resolution are important requirements in many acoustic focusing applications. In this sense, acoustic systems with dynamic control of the focus are advantageous over devices with a fixed focal length, since they can be adapted to operate in various application scenarios. Nowadays, conventional methods to control the focal length are mainly based on either changing the geometric profile of the lens^16^, or modifying the properties of liquid crystals by applying an electric field^17^. On the other hand, the devices capable of providing subwavelength resolution usually have a significant diameter of at least several tens of wavelengths, which results in bulky acoustic systems. With the introduction of acoustic jets, subwavelength focusing is now feasible even with lenses with mesoscale dimensions (diameter to wavelength ratio lower than ten). Mesoscale lenses involve the interaction of acoustic waves with structures of intermediate scale, which are too small to be described by traditional continuum methods. However, there are still no available systems which can provide at the same time both narrow focusing and dynamic control of the focal length while keeping compact dimensions in terms of the wavelength. Note that in reference^8^ the diameter of the 2D lens was significant and did not meet the mesoscale requirement. In this work, we present a spherical lens with a diameter of 6.7*λ* capable of achieving subwavelength focusing with continuous control of the focal distance and both longitudinal and lateral resolutions.

The spherical lens consists of a hollow container filled with an inner liquid with certain acoustic properties. The sound speed contrast between the inner liquid and the host medium provides the focusing profile of the spherical lens. Thus, the lens focal length can be shifted by replacing the lens inner liquid with another one with different acoustic properties. However, this procedure requires to stop the focusing application during a certain amount of time while the lens is being emptied, cleaned and refilled with the new inner liquid. Alternatively, a more feasible solution would be to have a set of spherical containers with different inner liquids that comprise a certain focal length range and could be faster exchanged. In any case, this procedure would only allow a discrete set of focal lengths and would not be a real configurable lens because it would still require the interruption of the current application in order to modify the focal length.

Most liquid lenses employed for varifocal imaging are able to control their focal distance by modifying their shape^18–20^. Here, we propose a different approach based on filling the spherical lens ABS container with two miscible liquids of different sound speeds. Depending on the mixing ratio between the volumes of both liquids, the sound speed of the liquid mixture can be accurately adjusted to any desired value inside a continuous range, resulting in a shift on the lens focal distance. Moreover, the procedure used to adjust the volume ratio between both liquids can be fully automatized by connecting an input and an output port to the hollow container and determining the type and amount of liquid that has to be introduced to either increase or reduce the sound speed of the liquid mixture, thus achieving an easy-to-implement and dynamic control mechanism to shift the focal distance. Our approach presents two main advantages over other implementations based on spherical lenses. First, it allows a continuous shifting of the lens focal distance and moreover, it can be implemented as a dynamic configurable procedure which does not require the interruption of the focusing application to modify the properties of the acoustic jet. Thus, this method provides an additional degree of flexibility for spherical lenses that has not been previously reported in literature to the best of our knowledge. Experimental measurements for ethanol-water liquid mixtures are presented, showing good agreement with numerical results and demonstrating the viability of this type of lenses.

## Results

An acoustic jet can be characterized by its focal distance (*F*) and its resolution in both the longitudinal and lateral directions, parameters which specify the location and shape of the jet^6^. In this paper, the focal distance is defined as the distance between the center of the spherical lens and the location of the maximum inside the focus. Longitudinal and lateral resolutions are determined by the full length half maximum (FLHM) and the full width half maximum (FWHM), respectively. Both FLHM and FWHM parameters measure the interval in which the acoustic intensity is reduced to half of its maximum value in their respective axes. All these three parameters depend on the refraction index of the inner liquid, which is defined as *n* = *c*_0_/*c*_*m*_, being *c*_0_ the sound speed of the host medium and *c*_*m*_ the sound speed of the inner liquid. As stated above, when two miscible liquids are used, the refraction index of the liquid mixture can be precisely and continuously controlled by varying the mixture volume ratio between both liquids.

The spherical shell of the lens has a thickness *t*_*h*_ ≪ *λ* and a diameter *D*. This container is filled with an inner liquid mixture with density *ρ*_*m*_ and sound speed *c*_*m*_. The lens is placed inside a water tank with density *ρ*_0_ and sound speed *c*_0_ at a distance *d* from a directional ultrasound transducer. The inner liquid used in this work is a mixture of ethanol and water. The mixture can be characterized by the volume fraction, *φ*, defined as

1 $$\phi =\frac{{V}_{w}}{{V}_{w}+{V}_{e}},$$

where *V*_*w*_ and *V*_*e*_ are the water an ethanol volumes used to produce the mixture, respectively.

**Figure 1:**
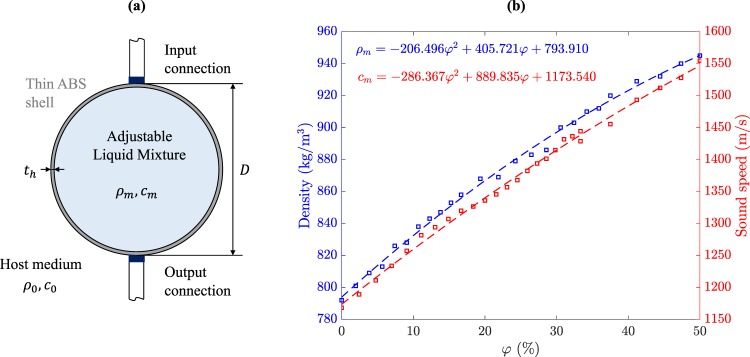
(**a**) Schematic concept of the fully automated lens and (**b**) acoustic properties (density in blue and sound speed in red) of the liquid mixture against the volume fraction (*φ*). Experiments (squares) and polynomial fits (dashed lines).

Figure 1(a) depicts a schematic concept of the fully automated spherical lens. As stated before, the mixing ratio between both liquids can be controlled by introducing the correct amount of one of the substances through the input connection and extracting the correct volume of the previous mixture through the output connection. Figure 1(b) shows the acoustic properties of the ethanol-water liquid mixture as a function of the volume fraction. This measures have been used to obtain second order polynomial fits depicted as dashed lines, which provide continuous characterization of the liquid mixture in the 0 < *φ* < 0.5 range. As it can be observed from Fig. 1(b), the sound speed of the liquid mixture is higher than that of both water and ethanol for certain *φ* values, ranging from 1174 to 1547 m/s for volume fractions between 0 and 50%.

**Figure 2:**
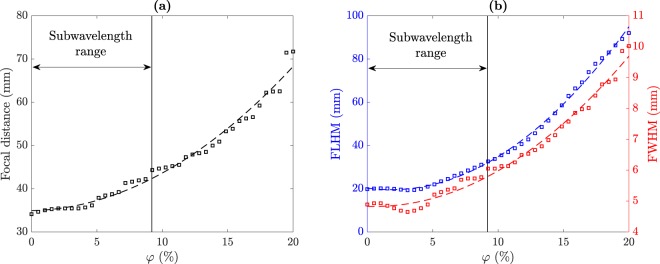
Simulated acoustic jet parameters vs. *φ*: (**a**) focal distance, (**b**) FLHM (blue) and FWHM (red).

In this work, the spherical lens has a diameter *D* = 40 mm and a shell thickness of *t*_*h*_ = 0.25 mm. The thickness of the spherical container is a parameter that affects the generation of the acoustic jet and can be optimized for a certain inner liquid using FEM simulations. However, when dealing with liquid mixtures, the optimum container thickness is different at distinct volume fractions, and therefore, the whole range of volume fractions should be considered to determine the overall optimum thickness. The working frequency is fixed to *f*_0_ = 250 kHz. Acoustic jets are generated using spherical lens with diameters between 3 and 30 wavelengths. Under this assumption, the working bandwidth of the proposed lens would be ranging from 110 kHz to 1.12 MHz, approximately. This operating bandwidth can be further shrunk due to additional limitations such as the emitter transducer bandwidth. Figure 2 shows the acoustic jet parameters as a function of the volume fraction computed using the FEM model described at the Methods section. The properties of the liquid mixture of the FEM model have been obtained from the polynomial fits depicted in Fig. 1(b). The volume fraction ranges from 0%, which corresponds to pure ethanol with a refraction index of *n*_*m*_ = 1.269, to 20% (*n*_*m*_ = 1.1). In Fig. 2, squares represent numerical results, whereas dashed lines represent the corresponding polynomial fits. Figure 2(a) depicts the focal distance for the spherical lens as a function of the filling fraction. As it can be observed, the focal distance follows a smooth quadratic tendency that increases with the *φ* parameter and allows a fine and continuous control of the focus location. Figure 2(b) depicts the longitudinal (blue) and lateral (red) resolutions against *φ*. As it can be observed from the figure, both parameters present a similar quadratic dependence on the *φ* parameter than that of the focal distance. This means that as the volume fraction increases, the resolution of the lens is reduced in both the longitudinal and the lateral directions. Moreover, Fig. 2(b) shows that if subwavelength resolution is required (FWHM < 5.96 mm), the volume fraction has to be lower than 9% approximately.

Therefore, the simulation results depicted in Fig. 2 can be used to obtain the required volume fraction in order to achieve a specific focal distance and resolution. With this information, an automated mechanism could be implemented to adjust the volume fraction of the mixture as shown in Fig. 1(a).

**Figure 3:**
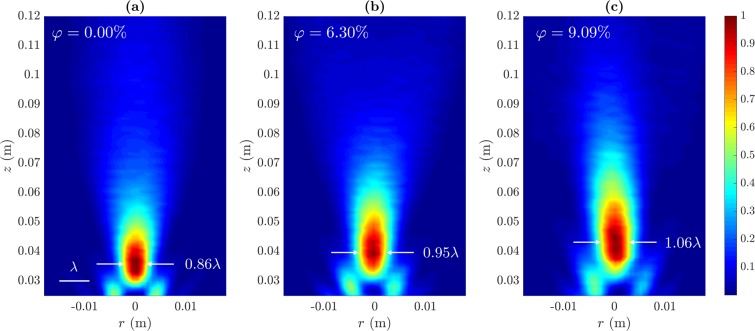
Measured normalized intensity maps: (**a**) *φ* = 0.00%, (**b**) *φ* = 6.30% and (**c**) *φ* = 9.09%.

Experimental results have been carried out in order to validate the proposed method. Figure 3 depicts the measured intensity maps for three different volume fractions. Figure 3(a) corresponds to pure ethanol (*φ* = 0.00%), while 3(b) and 3(c) correspond to *φ* = 6.30% and *φ* = 9.09%, respectively. Sound speed and density values for these volume fractions have been obtained from the polynomial fits shown in Fig. 1. Each map is normalized to its maximum intensity value. As it can be observed, there is a focal displacement in the acoustic jet, and the focal distance increases with the *φ* parameter. Moreover, the focal area spreads out in both longitudinal and lateral directions, as expected from the simulation results shown in Fig. 2.

**Figure 4:**
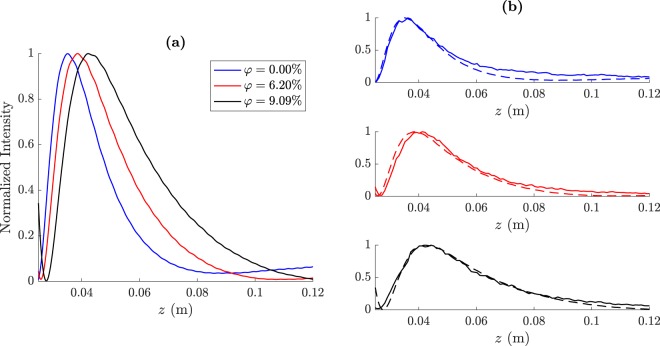
Focusing profiles at *r* = 0: (**a**) simulation results and (**b**) experimental measurements (solid lines) compared to their simulated counterparts (dashed lines).

Figure 4 depicts the focusing profiles for the three volume fractions that have been previously considered. Figure 4(a) shows the three computed focusing profiles for comparison purposes, while Fig. 4(b) shows three separated plots with the experimental measurements (solid lines) and the simulations results (dashed lines) at each volume fraction. As it can be observed from Fig. 4, the pure Ethanol lens with *φ* = 0% provides the shortest focal distance with the highest longitudinal resolution, while the ethanol-water mixture with *φ* = 9.09% achieves the longest focal distance with the lowest resolution. The *φ* = 6.30% configuration provides intermediate values for both focal distance and longitudinal resolution.

**Figure 5:**
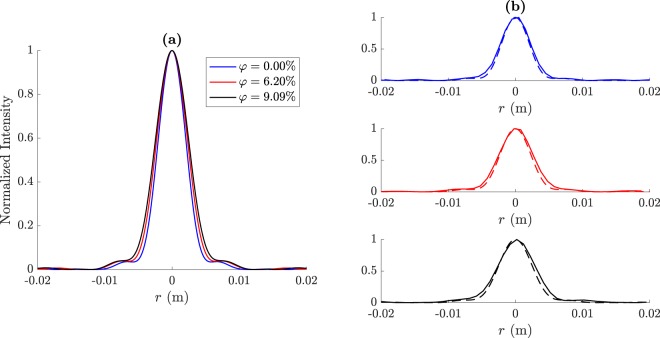
Transversal intensity cuts at *z* = *F*: (**a**) simulation results and (**b**) experimental measurements (solid lines) compared to their simulated counterparts (dashed lines).

Figure 5 depicts the transversal intensity cuts at *z* = *F* for the three volume fractions that have been previously considered. Figure 5(a) shows the three computed transversal cuts for comparison purposes, while Fig. 5(b) shows three separated plots with the experimental measurements (solid lines) and the simulations results (dashed lines) at each volume fraction. As it can be observed from Fig. 5, the pure ethanol lens (*φ* = 0%) achieves the highest lateral resolution providing the narrowest focal spot with a measured FWHM of 5.1 mm, which corresponds to 0.856*λ*. The *φ* = 6.30% lens provides a measured lateral resolution of 5.638 mm (0.946*λ*). Finally, the *φ* = 9.09% configuration shows the lowest resolution, with a FWHM of 6.293 mm (1.056*λ*). These experimental results agree with the numerical simulations, and demonstrate that spherical lenses filled with ethanol-water mixtures can provide subwavelength resolution for a water volume ratio lower than 9%, achieving a continuously tunable focal distance ranging from 35 to 43 mm.

Table 1 shows the acoustic jet parameter simulation and experimental values for the three volume fractions previously considered. As it can be observed from Fig. 5 and Table 1, FEM results are in good agreement with experimental measurements. The lowest focal distance is achieved when the spherical lens is filled with pure ethanol (*φ* = 0%), and corresponds to a measured value of *F* = 35.25 mm or *F* = 5.91*λ*. The focal distance is shifted to 40.25 and 44.25 mm, when the volume fraction is modified to *φ* = 6.30% and *φ* = 9.09%, respectively. Lateral and longitudinal resolutions also exhibit similar tendencies with *φ*, increasing their value quadratically as the volume fraction augments and showing a linear dependence with the focal distance. Lateral resolution is below the wavelengh for both *φ* = 0% and *φ* = 6.30% achieving FWHM values of 0.86*λ* and 0.95*λ*, respectively. These results demonstrate that the dynamic control of the liquid mixture sound speed allows a continuous shift of the focal length, while the lateral resolution can be kept below the wavelength for a certain focal length range.

**Table 1:** Measured (EXP) and simulated (FEM) acoustic jet parameters for different *φ* values.

*φ* (%)		*F* (mm)	*F* (*λ*)	FLHM (mm)	FLHM (*λ*)	FWHM (mm)	FWHM (*λ*)
0.00	EXP	35.25	5.91	20.61	3.46	5.10	0.86
	FEM	34.59	5.80	20.03	3.36	4.91	0.82
6.30	EXP	40.25	6.75	26.03	4.37	5.64	0.95
	FEM	38.83	6.52	24.91	4.18	5.39	0.90
9.09	EXP	44.25	7.42	31.45	5.28	6.29	1.06
	FEM	42.80	7.18	32.00	5.37	5.95	1.00

## Discussion

Spherical lenses filled with liquid mixtures provide a continuous and dynamic control mechanism to adjust the lens focal distance, while maintaining a good lateral resolution. By shifting the volume ratio between both liquids, all acoustic jet parameters can be accurately tuned. A configurable spherical lens filled with ethanol and water has been experimentally characterized, measuring its acoustic intensity map for three different volume fractions. Simulation and experimental results show good agreement, demonstrating the performance of these lenses, which present a significant advantage over conventional single liquid spherical lenses, and are very appealing for many acoustic applications, such as acoustic microscopy. The lateral resolution is kept below the wavelength for a certain volume fraction range between 0% and 9%. This lateral resolution could be further increased, going beyond the diffraction limit, by selecting two different miscible inner liquids, such as ethyl iodide and ethanol, with the requirement that one of these liquids (ethyl iodide) presents a higher sound speed contrast with the host medium. Numerical simulations show that a pure ethyl iodide lens can achieve a lateral resolution of 0.42*λ*, which is beyond the diffraction limit. Thus, this work opens new possibilities to implement higher resolution compact acoustic systems with continuous tunability of the main focusing parameters of the jet.

## Methods

### Numerical model

**Figure 6:**
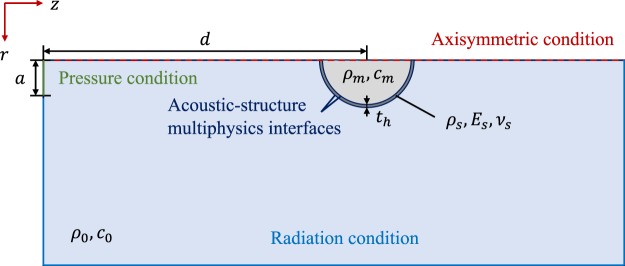
Schematic of the FEM model geometry and boundary conditions.

The acoustic pressure generated by the spherical lens has been numerically calculated using a Finite Element Method (FEM) model and the commercial software COMSOL Multiphysics. The numerical model is implemented as a 2D axisymmetric problem, because both transducer and spherical lens present rotation symmetry. The spherical container is made of ABS plastic with thickness *t*_*h*_ = 0.25 mm and diameter *D* = 40 mm. The directional transducer has been implemented as a pressure condition at a distance *d* = 340 mm from the ABS sphere with *a* = 15 mm being half the active diameter of the transducer. The working frequency is fixed to *f*_0_ = 250 kHz, which provides a diameter to wavelength ratio of *D*/*λ* = 6.7. A radiation condition is established at the boundaries in order to emulate the Sommerfeld radiation condition and avoid reflections. In order to consider the elastic properties of the solid ABS shell, the FEM model implements the acoustic-structure multiphysics interface. ABS has been modelled as a linear elastic material characterized by its Young modulus (*E*_*s*_), Poisson ratio (*ν*_*s*_) and density (*ρ*_*s*_). Figure 6 depicts a schematic diagram of the FEM model and Table 2 shows the properties of the different materials used in the simulation.

**Table 2:** Properties of the spherical lens materials.

Material	Properties
Water	*ρ*_0_ = 1000 kg/m^3^, *c*_0_ = 1490 m/s
Ethanol	*ρ*_*m*_ = 793 kg/m^3^, *c*_*m*_ = 1174 m/s
ABS	*ρ*_*s*_ = 1050 kg/m^3^, *E*_*s*_ = 3 GPa, *ν*_*s*_ = 0.35

### Experimental set-up

**Figure 7:**
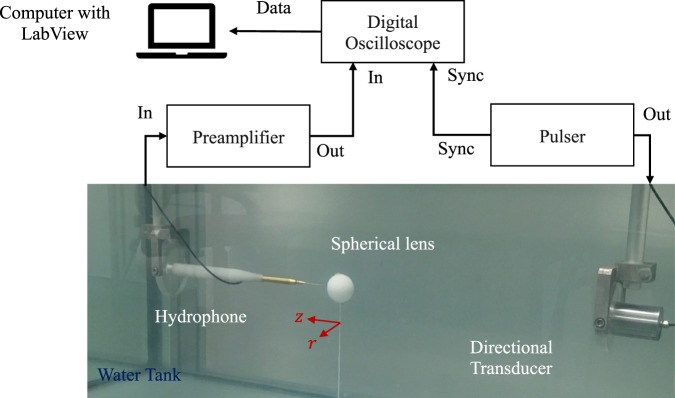
Experimental set-up.

Experimental measurements have been carried out in order to validate the numerical results. The experimental set-up consists of a 3D automated positioning system immersed in a 1 × 0.5 × 0.5 m^3^ tank filled with distilled water. A needle hydrophone with a diameter of 1.5 mm and −4 dB bandwidth ranging from 200 kHz to 25 MHz is employed as receiver. The hydrophone is fixed to a programmable robotic arm, which can move in any of the three spatial directions with a resolution of 1 × 1 × 1 mm^3^. An Imasonic directional transducer with 30 mm of active diameter and a working frequency of 250 kHz is used as emitter. Figure 7 shows a picture of the experimental set-up. The spherical lens is attached to a filament to keep it in place.

## References

[1] Lu YF, Zhang L, Song WD, Zheng YW, Luk’yanchuk BS (2000). Laser writing of a subwavelength structure on silicon (100) surfaces with particle-enhanced optical irradiation. J. Exp. Theor. Phys. Lett..

[2] Chen Z, Taflove A, Backman V (2004). Photonic nanojet enhancement of backscattering of light by nanoparticles: a potential novel visible-light ultramicroscopy technique. Opt. Express.

[3] Heifetz A, Kong S-C, Sahakian AV, Taflove A, Backman V (2009). Photonic Nanojets. J. Comput. Theor. Nanosci..

[4] Thomas DC, Gee KL, Turley RS (2009). A balloon lens: Acoustic scattering from a penetrable sphere. Am. J. Phys..

[5] Parrales Borrero MA, Pérez-Saborid M, Fernández García JM (2011). Acoustic scattering from a spherical lens irradiated by a finite transducer: Focusing effect and refraction. Am. J. Phys..

[6] Minin OV, Minin IV (2017). Acoustojet: acoustic analogue of photonic jet phenomenon based on penetrable 3D particle. Opt. Quantum Electron..

[7] Lopes JH (2017). Focusing Acoustic Beams with a Ball-Shaped Lens beyond the Diffraction Limit. Phys. Rev. Appl..

[8] Veira Canle D (2019). Practical realization of a sub-l/2 acoustic jet. Sci. Reports.

[9] Chen J, Xiao J, Lisevych D, Shakouri A, Fan Z (2018). Deep-subwavelength control of acoustic waves in an ultra-compact metasurface lens. Nat. Commun..

[10] Assouar B (2018). Acoustic metasurfaces. Nat. Rev. Mater..

[11] Al Jahdali R, Wu Y (2016). High transmission acoustic focusing by impedance-matched acoustic meta-surfaces. Appl. Phys. Lett..

[12] Jiménez-Gambín, S., Jiménez, N., Benlloch, J. M. & Camarena, F. Holograms to focus arbitrary ultrasonic fields through the skull. 1902.06716 (2019).

[13] Hyun J (2018). Realization of an ultrathin acoustic lens for subwavelength focusing in the megasonic range. Sci. Reports.

[14] Chen J, Rao J, Lisevych D, Fan Z (2019). Broadband ultrasonic focusing in water with an ultra-compact metasurface lens. Appl. Phys. Lett..

[15] Chen J, Sun Z, Fan Z (2019). Groove-structured meta-surface for patterned sub-diffraction sound focusing. Appl. Phys. Lett..

[16] Oku H, Hashimoto K, Ishikawa M (2004). Variable-focus lens with 1-kHz bandwidth. Opt. Express.

[17] Honma M, Nose T, Yanase S, Yamaguchi R, Sato S (2009). Liquid-crystal variable-focus lenses with a spatially-distributed tilt angles. Opt. Express.

[18] Gorman CB, Biebuyck HA, Whitesides GM (1995). Control of the Shape of Liquid Lenses on a Modified Gold Surface Using an Applied Electrical Potential across a Self-Assembled Monolayer. Langmuir.

[19] Berge B, Peseux J (2000). Variable focal lens controlled by an external voltage: An application of electrowetting. The Eur. Phys. J. E.

[20] López CA, Hirsa AH (2008). Fast focusing using a pinned-contact oscillating liquid lens. Nat. Photonics.

